# Upregulation of chemokine receptor CCR10 is essential for glioma proliferation, invasion and patient survival

**DOI:** 10.18632/oncotarget.2134

**Published:** 2014-06-26

**Authors:** Lingchao Chen, Xing Liu, Hai-Yan Zhang, Wenzong Du, Zhiyong Qin, Yu Yao, Ying Mao, Liangfu Zhou

**Affiliations:** ^1^ Department of Neurosurgery, Huashan Hospital, Fudan University, Shanghai, China; ^2^ Department of Neurosurgery, the Second Affiliated Hospital of Harbin Medical University, Harbin, China; ^3^ Department of Obstetrics and Gynecology, International Peace Maternal and Children's Hospital, Shanghai Jiaotong University, Shanghai, China

**Keywords:** CCR10, Glioma, Akt pathway

## Abstract

Human gliomas are characterized by their invasion of normal brain structures irrespective of their grade of malignancy. Tumor cell invasion share many similarities with leukocyte trafficking, which is critically regulated by chemokines and their receptors. Here we report that the chemokine receptor CCR10 is highly expressed in human glioblastoma compared with control brain tissue. *In vitro*, signaling through CCL27-CCR10 mediates activation of p-Akt, and subsequently induces proliferation and invasive responses. Cell proliferation and invasion promoted by CCL27 were blocked by inhibition of p-Akt or CCR10. *In vivo*, down-regulation of CCR10 significantly impairs growth of glioma. Clinically, High CCR10 expression in GBM correlated with p-Akt, shorter overall survival and progression-free survival (P < 0.05). Together, these findings suggest that elevated CCR10 is a critical molecular event associated with gliomagenesis.

## INTRODUCTION

Glioblastoma is the most malignant primary brain tumor in human with poor survival despite multimodality treatment [1-3]. In this tumor, malignant invasion was one of the most principal hallmarks [3]. Although a number of molecules have been implicated in the invasion of glioma [4-8], the precise mechanisms determining the directional migration and invasion of tumour cells remain to be established.

Chemokine receptors are cytokine receptors found on the surface of certain cells that interact with a type of cytokine called a chemokine. There have been 19 distinct chemokine receptors in mammals. Each has a 7-transmembrane structure and couples to G-protein for signal transduction within a cell, making them members of a large protein family of G protein-coupled receptors. Following interaction with their specific chemokine ligands, chemokine receptors trigger a flux in intracellular calcium (Ca2+) ions (calcium signaling). This causes cell responses, involved in leukocyte migration, angiogenesis, hematopoiesis and tumor developments[9-11]. In addition, they may also regulate tumor growth, survival, proliferation and migration of cancer cells either directly by transformation or indirectly by enhancing angiogenesis or recruiting leukocytes [10-16].

So far, various chemokine receptors are expressed on plenty of cancers, including glioma[10, 11, 13, 17, 18]. Numerous studies of their role in tumor development are emerging, such as CXCR4, and CXCR7 [19, 20]. Knowledge of chemokines network and their contribution to glioma progression may lead to a new therapeutic approach and more effective therapy. CCR10 is a chemokine receptor that in humans is encoded by the *CCR10* gene. Its ligands are CCL27 and CCL28. CCR10 is normally expressed by melanocytes, plasma cells and skin-homing T cells. Melanoma cell transduction of CCR10 significantly increases the development of lymph node metastasis in mice after inoculation in the skin. Besides, CCR10-CCL27 interactions also play a key role in T cell homing in inflamed skin and melanoma directing metastasis. However, the role of CCR10 in glioma was not well known. In this study, we report that CCR10 is highly expressed in human glioblastoma and correlated with shorter overall survival and progression-free survival. Signaling through CCL27-CCR10 mediates activation of p-Akt, and subsequently induces proliferation and invasive responses. These results suggest that CCR10 high expressed in glioma is essential for tumor proliferation, invasion and progression.

## RESULTS

### CCR10 is highly expressed in glioblastomas

**Figure 1:**
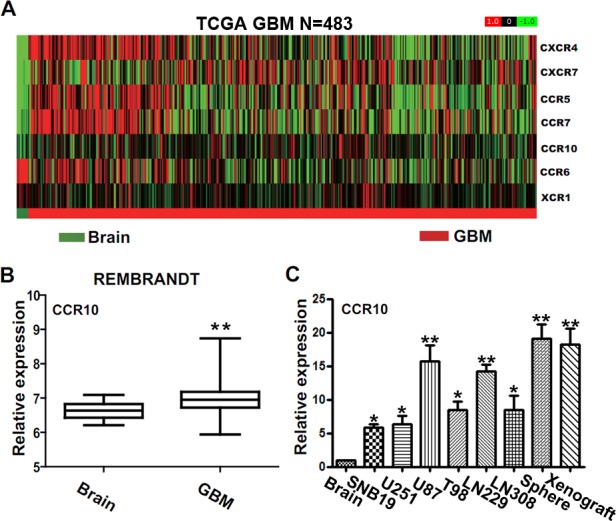
The expression of CCR10 in glioma tissues (A) Heat maps of differentially expressed chemokine receptors in the normal brain tissue and GBM samples from TCGA database (N=483). (B) The mRNA expression of CCR10 in control brain and GBM in Rembrandt database. (C) Real-time PCR analyzed the mRNA expression of CCR10 in normal brain and glioma cell lines.

Chemokine receptors are well known for tumor proliferation and invasion. Here we use GBM gene expression profile from TCGA compared chemokine receptors with normal brain tissue. There are five chemokine receptors overexpressed in GBM, including CXCR4, CXCR7, CCR5, CCR7 and CCR10 (Figure 1A). We focused on CCR10, which was known for tumor development in plenty of cancers [22, 23]. However, little work was done about CCR10 in GBM. Strikingly, in an independent set of 227 human GBM tumors from Rembrandt, GSE16011 and oncomine, the expression of CCR10 in GBM was also higher than normal brain tissues (Figure 1B and Supplementary Figure 1-2). Consistent with the expression data, we found robust expression of CCR10 in 6 glioma cells, neurosphere and xenograft models at levels 5.81–19.13 folds higher than the levels observed in a control brain tissue (Figure 1C). Furthermore, IHC assay demonstrated CCR10 expression was significantly higher in the glioma samples than in the control brain tissues. A gradually stronger CCR10 expression was found from grade II to grade IV samples and cell lines (Supplementary Figure 3).

### CCL27 promote glioma cell proliferation and invasion

**Figure 2:**
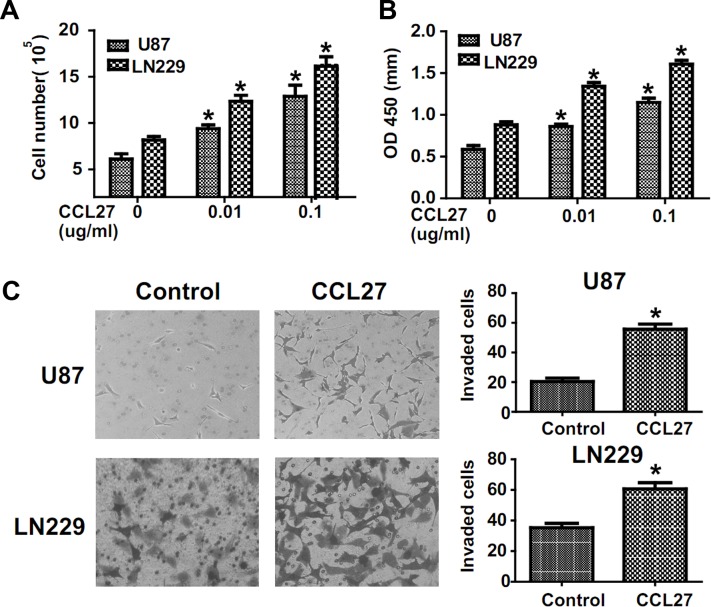
CCR10 activation promote proliferation and invasion *in vitro* (A-B) Representative histogram showing the numbers of cells and OD value by CCL27 treated cells. (C) Transwell assays indicate increased invasion in U87 and LN229 cells following CCL27 treatment.

To assess effects of CCL27 on glioma cells, we stimulated U87 and LN229 cells with recombinant human chemokine CCL27 under serum deprivation conditions and examed cell proliferation by cell count and MTT assay. Cell proliferation was increased by stimulation with CCL27 in a dose-dependent manner (Figure 2A-B). Moreover, we chose transwell assay and wound healing assay to assess the role of CCL27 in invasion. Exogenous CCL27 stimulation increased the invasion of glioma cells compared with control (Figure 2C and supplementary Figure 4). Together, these data demonstrates that CCR10 activation impacts proliferation and invasion of glioma *in vitro*.

### Involvement of p-Akt in CCR10 mediated proliferation and invasion

After engagement with appropriate ligands, chemokine receptors trigger a complex cascade of intracellular signaling events that activate downstreem pathway. To identify the downstream pathways that may contribute to CCL27-CCR10 signaling, we performed gene expression profiling to compare the CCL27 high/low and CCR10 high/low GBM samples. Analyses of biological pathways using gene-set enrichment analysis (GSEA) [24] revealed that pathways significantly (P <0.05) upregulated in the CCL27 or CCR10 GBM samples included SRC and PDGF signal gene set (Supplementary Figure 5). Because SRC and PDGF are key upstream mediators of PI3K/Akt signaling pathway, and has been shown to have important roles in cell proliferation, migration and survival [25, 26], we hypothesized that Akt activation might contribute to CCR10 mediated proliferation and invasion in glioma.

**Figure 3:**
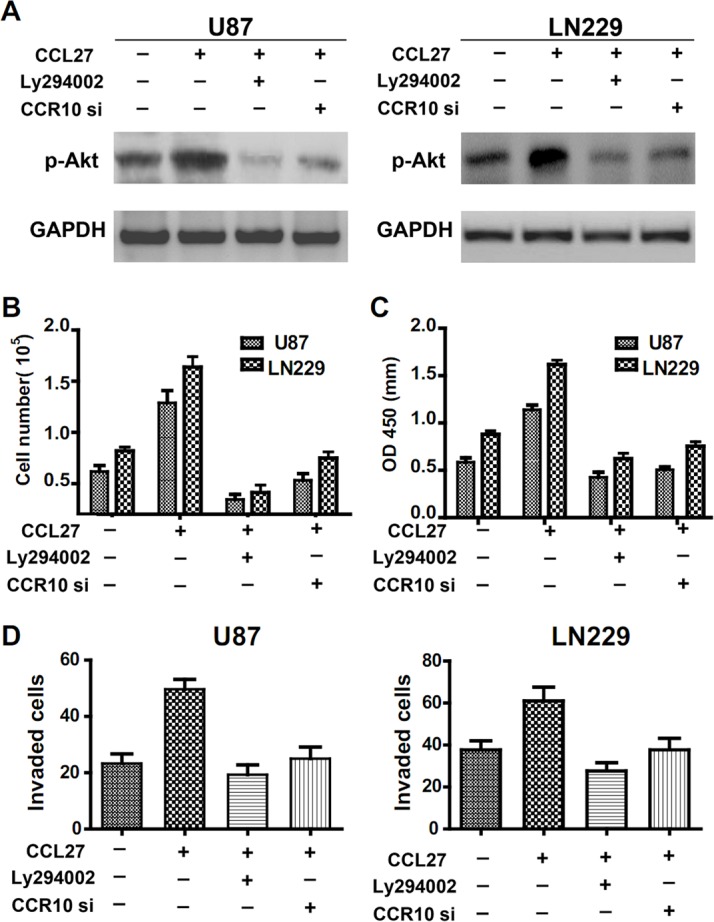
Involvement of p-Akt in CCR10 mediated proliferation and invasion (A) p-Akt expression levels in U87 and LN229 cells treated with CCL27, LY294002 or CCR10 siRNA were assessed by western blot. (B-D) Representative cartogram showing the numbers of cells, OD value and invaded cells by CCL2, LY294002 or CCR10 siRNA treated cells.

After administration of CCL27 to glioma cells, we observed a strong increase in p-Akt. The expression of p-Akt was nearly completely inhibited by selective PI3K inhibitors LY294002 (Figure 3A). To knockdown CCR10, we use siRNA method. Multiple CCR10 siRNA and RNAi resistant version of CCR10 have been tested to exclude off-target effects. CCR10 siRNA_1 was more effective than other siRNA (Supplementary Figure 6). Thus, we use siRNA_1 in the following study. The expression of p-Akt was also inhibited by treatment with CCR10 siRNA in the presence of CCL27 (Figure 3A). To demonstrate the significance of PI3K/Akt signaling in CCL27-mediated proliferation and invasion, CCL27 treated U87 and LN229 cells were exposed in the absence or presence of CCR10 siRNA or LY294002. As before, administration of CCL27 alone increased proliferation. When LY294002 was added, the cell counts and OD value were significantly reduced (Figure 3B). Treatment of CCR10 siRNA also suppressed the proliferation of glioma in the presence of CCL27. In additional, similar effect of LY294002 and CCR10 siRNA were observed in transwell assay (Figure 3C). Therefore, p-Akt was involved in CCL27/CCR10 mediated proliferation and invasion of glioma *in vitro*.

### CCR10-neutralization inhibits growth *in vivo*

**Figure 4:**
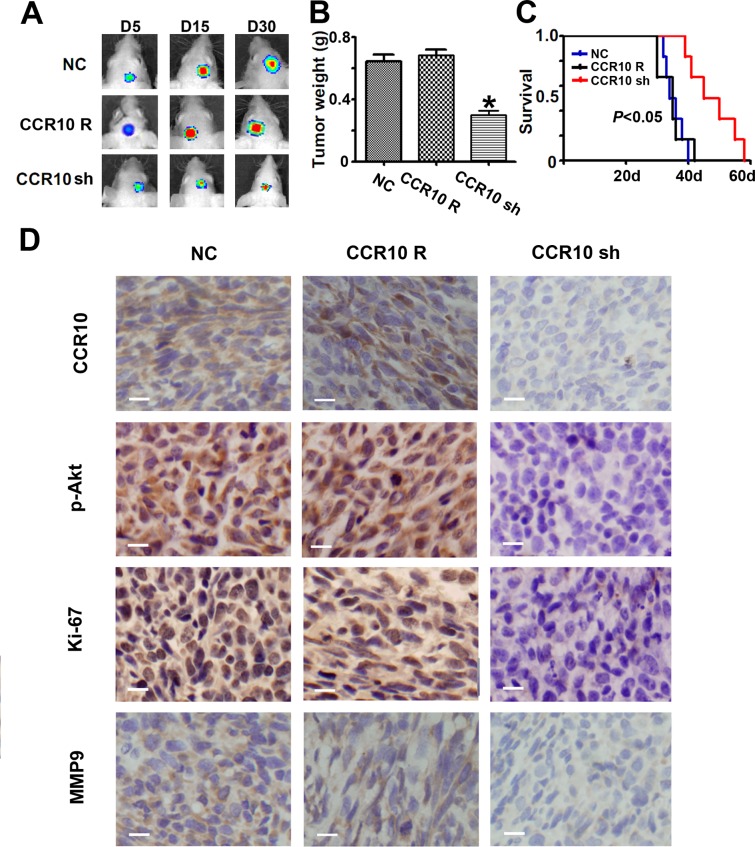
Down-regulation of CCR10 inhibits growth *in vivo* (A) Luminescence imaging for CCR10 shRNA treated LN229 tumors vs. CCR10 shRNA resistant and scramble-treated controls. (B) Tumors mass was determined in CCR10 shRNA treated LN229 tumors vs. CCR10 shRNA resistant and scramble-treated controls. (C) Survival analysis was determined in CCR10 shRNA treated LN229 tumors vs. CCR10 shRNA resistant and scramble-treated controls. (D) Representative photomicrographs of tumor sections following IHC analysis for CCR10, p-Akt, Ki-67 and MMP9 expression.

Having established that CCR10 activation promote proliferation and invasion *in vitro*, we evaluated the contribution of CCL27/CCR10 signaling on tumor growth *in vivo* by using experimental intracranial models. Since CCR10 shRNA was more effective in LN229 than U87, we chose LN229 cell for the *in vivo* models. When the mice intracranially transplanted with LN229 that stably express luciferase and CCR10 shRNA, CCR10 shRNA resistant or control vector, bioluminescence imaging was done for the whole body. CCR10 shRNA treated LN229 cells displayed a marked reduction of the tumor (Figure 4A-B). To analyze the survival times of the treatment groups, we generated Kaplan-Meier survival curves (Figure 4C), which demonstrated that CCR10 shRNA significantly prolonged survival compared with CCR10 shRNA resistant and control group. Further, CCR10 shRNA treatment displayed decreased expression of CCR10 and p-Akt (Figure 4D), which was consistent with the *in vitro* results. In addition, MMP9 and Ki67, markers of tumor invasion and proliferation were also suppressed by CCR10 shRNA (Figure 4D). These data indicates that knockdown of CCR10 expression inhibits glioma growth *in vivo*.

### Increased CCR10 expression correlates with poor survival in human glioma

**Figure 5:**
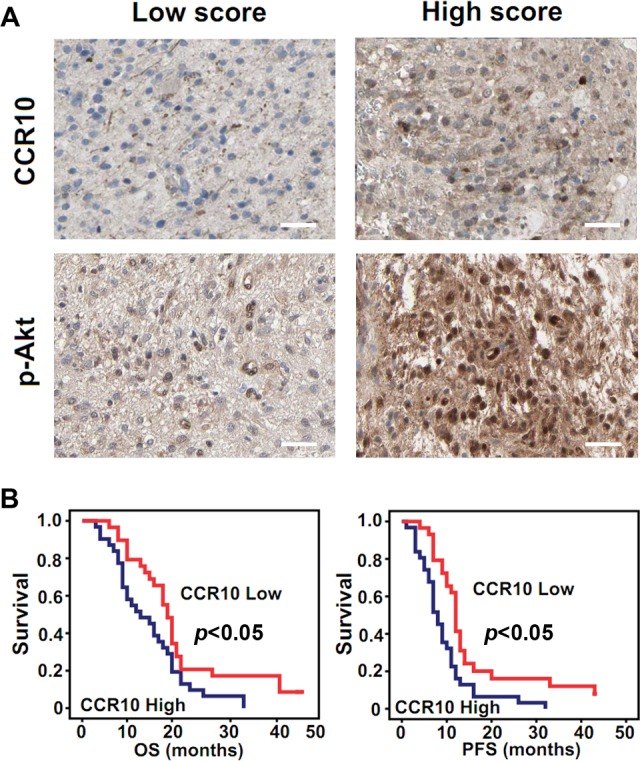
CCR10 expression positively correlate with p-Akt and correlated with poor survival in GBM (A) Expression of CCR10 and p-Akt in resected glioma specimen was assessed by IHC assay. (B) Kaplan–Meier survival curves indicating cumulative survival as a function of time for those patients with CCR10 high expression versus low expression. The patients with high CCR10 expression experience significantly worse outcome.

To determine whether the positive correlation between CCR10 and p-Akt expression was consistent *in vitro* and in patient samples, we quantified expression levels of CCR10 and p-Akt in 60 GBM tissue specimens by IHC assay (Supplementary Table 1). High scores CCR10 glioma contained comparatively high p-Akt expression than those in low scores specimen (Figure 5A). Pearson's chi-squared test demonstrated that CCR10 in tumor tissues positively correlated with p-Akt expression (Table 1). These data suggested that increased p-Akt expression might result from CCR10 over-expression in human glioma. Retrospective analysis of the clinical outcome associated with each tissue specimen revealed that reduced immune detection of CCR10 correlated with long OS and PFS (Figure 5B and Table 2). In summary, these data indicate that CCR10 regulation of p-Akt expression has significantly clinical impact on glioma.

**Table 1:** Summary of CCR10 and p-Akt immunohistochemical staining in tumor tissue sample from 60 GBM

		p-Akt		Total
		High	Low	
CCR10	High	29(93.5%)	2(6.5%)	31
	Low	5(17.2%)	24(82.8%)	29
Total		34	26	60

Pearson's chi-squared test was used to analyze for significance of the relationship between CCR10 and p-Akt expression (p<0.05).

**Table 2:** Multivariate Cox regression analyses for OS of 60 GBM patients

	Multivariate	
variables	HR (95% CI)	P value
Ki-67	1.39(0.796-2.431)	0.247
Kps	0.656(0.378-1.139)	0.134
CCR10	1.766(1.03-3.026)	0.039

## DISCUSSION

Accumulating evidence suggests that chemokines and their receptors interaction may drive tumor growth and invasion. Human gliomas are characterized by their invasion of normal brain structures. If this is the case, then the development of strategies to effectively target the chemokines receptors will be required to improve therapeutic outcome. We describe such an approach based on comprehensive analysis of all chemokine receptors and clinical data in TCGA. After compared the differential gene expression between normal brain and GBM, we identified five chemokine receptors, including CXCR4, CXCR7, CCR5, CCR7 and CCR10. Among the 5 upregulated receptors, we focused on CCR10, which was known for tumor development in plenty of cancers [22, 23, 27-31]. However, little work was done about CCR10 in GBM. Consistent with the TCGA data, Rembrandt data and qRT-PCR assay confirmed the robust expression of CCR10 in glioma tissue and cell lines. Thus, we chose CCR10 for the following function study.

CCR10 is a chemokine receptor that in humans is encoded by the *CCR10* gene. Its ligands are CCL27 and CCL28. B16 melanoma cell transduction of CCR10 significantly increases the development of lymph node metastasis in mice after inoculation in the skin [27]. Weinlich G. et al. compared the potential role of the chemokine receptors in human primary cutaneous melanoma and found that CCR7 and CCR10 over-expressions were found to be associated with a worse outcome of disease course independent of Breslow's tumor thickness and Clark level [31]. In some authors' experience, high expression levels of CCR10 in the skin and the high incidence of skin metastases indicate the importance of chemokine receptors in metastasis at this site [32, 33]. In our study, we demonstrated that CCR10 activation by CCL27 stimulation increased cell proliferation and invasion. *In vivo*, CCR10 shRNA treated LN229 cells displayed a marked reduction of the tumor. Kaplan-Meier survival analysis demonstrated that CCR10 shRNA significantly prolonged survival of glioma in NOD/SCID mice. Taken together, CCR10 may also play an important functional role in increasing the ability of neoplastic cells to grow and invade tissue in glioma.

Intracellular signaling by chemokine receptors is dependent on neighbouring G-proteins. Several signaling molecules related to the activation of chemokine receptors have been identified and include AKT/PKB, ERK1/2 mitogen-activated protein kinase (MAPK), phosphoinositide 3-kinase (PI3K), STAT3, and NF-kB. However, little is known about downstream signaling pathways regulated by CCL27-CCR10 interaction in glioma. GSEA is a computational method developed at the Broad Institute of MIT and Harvard that determines whether an a priori defined set of genes shows statistically significant, concordant differences between two biological states[24]. After collected the CCL27 high/low and CCR10 high/low GBM samples from TCGA, we analyzed of biological pathways using GSEA and found that SRC and PDGF signal gene set significantly (P <0.05) upregulated in the CCL27 and CCR10 GBM samples. SRC and PDGF are well known upstream mediators of PI3K/Akt signaling pathway, and has been shown to have important roles in cell proliferation, migration and survival [25, 26]. After mechanism validation both *in vitro* and *in vivo*, we demonstrated that p-Akt was key downstream signal involved in CCL27/CCR10 mediated proliferation and invasion of glioma. Finally, we also certified that CCR10 in tumor tissues positively correlated with p-Akt expression in GBM clinical samples. In addition, we analyzed the CCR10 expression in TCGA GBM four subtypes and found that CCR10 high expressed GBM was majorly distributed in non-proneural subtype (supplementary figure 7). Thus, CCR10 target therapy might be benefited for the non-proneural subtype GBM patients.

In summary, we show here that CCR10 is expressed in glioma and found that CCR10 expression correlates with poor survival of GBM patients. We propose the linkage between CCR10 and p-Akt, both of which play critical roles in carcinogenesis, has advanced our knowledge of how CCR10-overexpressing cancer cells mediated the glioma malignant.

## MATERIALS AND METHODS

### Cell culture and tissue samples

Human SNB19, U251, U87, T98, LN229 and LN308 glioblastoma cells were obtained from the China Academia Science Cell Repository, Shanghai, China. The cells were maintained in Dulbecco's modified Eagle's medium (Gibco, Los Angeles, CA, USA) supplemented with 10% fetal bovine serum (Gibco), and were incubated at 37°C in a 5% CO_2_ atmosphere. Cell transfection was performed using Lipofectamine 2000 (Invitrogen) according to the manufacturer's instructions.

80 paraffin-embedded gliomas specimens with clinical data were collected from Department of Neurosurgery, Huashan Hospital, Fudan University, including 10 grade II tumors, 10 grade III tumors and 60 grade IV tumors according to WHO classification (2007). 8 control brain tissues were obtained from the patients with severe traumatic brain injury (TBI) who needed post-trauma surgery. This study was approved by The Local Ethical Review Board of the Huashan hospital, Fudan University and written informed consent was obtained from all patients.

### Oligonucleotides and transfection

CCR10 siRNA and CCR10 shRNA were designed by BLOCK-iT™ RNAi Designer, invitrogen, USA. siRNA_1: Sense: GCUGGAUACUGCCGAUCUA; Antisense: UAGAUCGGCAGUAUCCAGC. siRNA_2: Sense: GCCUCAAUCCCGUUCUCUA; Antisense: UAGAGAACGGGAUUGAGGC. shRNA_91_top Insert Sequence: CACCGCTTTGCTACAAGGCCGATGTCGA AACATCGGCCTTGTAGCAAAGC; shRNA_91_bottom Insert Sequence: AAAAGCTTTGCTACAAGGCCGATGTTT CGACATCGGCCTTGTAGCAAAGC. Cells were transfected with CCR10 siRNA or scrambled oligonucleotides and Lipofectamine 2000 (Invitrogen) for vitro study. Cells were transfected with CCR10 shRNA or control vector and Lipofectamine 2000 (Invitrogen) for vivo study.

### Peptides and inhibitors

Recombinant human chemokine CCL27 were from Sino Biological Inc, added to cell cultures at a final concentration of 0.01 to 0.1μg/ml. PI3K inhibitor LY294002 (Sigma Chemical Co., St. Louis, MO) was dissolved in DMSO at a stock concentration of 10 mM and added to cell cultures at a final concentration of 30 μM.

### Proliferation assays by cell count and MTT assay

Following the transfection of LN229 and U87, cell count and MTT assay was used. Cells were counted using the Coulter Counter (Beckman Coulter). MTT assay was done as previous study [4, 21]. Each experiment was performed in triplicate. The absorbance values of each well were measured with a microplate spectrophotometer (Molecular Devices; Sunnyvale, CA) at 490 nm.

### *In Vitro* Invasion Assays

Invasion was measured using 24-well BioCoat cell culture inserts (BD Biosciences) with an 8-μm-porosity polyethylene terephthalate membrane coated with Matrigel Basement Membrane Matrix (100 μg/cm^2^). Briefly, the Matrigel was allowed to rehydrate for 2 h at 37C by adding warm, serum-free DMEM. The wells of the lower chamber were filled with medium containing 5% fetal bovine serum. U87 and LN229 Cells (5 × 10^4^) were seeded in the upper compartment (6.25-mm membrane size) in serum-free medium. Exogenous CCL27 (0.1μg/ml) were added in the upper chamber. After 24h incubation, non-invading cells were removed from the top well with a cotton swab, and bottom cells were fixed with 3% paraformaldehyde, stained with 0.1% crystal violet, and photographed in 9 independent 10× fields for 3 wells. Fold invasion was calculated relative to blank control. Data represents mean ± standard error (SE) of 3 independent experiments.

### Western blot and immunohistochemistry

Western blot and immunohistochemistry assay were performed as previously described [21]. Immunoblot and immunohistochemistry assays were performed using antibodies against CCR10 (1:1000 dilution, Santa Cruze, USA), p-Akt S473 (1:1000 dilution, Abcam, USA) and GAPDH (1:1000 dilution, Santa Cruze, USA). IHC scores were performed using a semiquantitative grading system as previous study [5]. Sections with no labeling or with <5% labeled cells were scored as 0. Sections were scored as 1 with labeling of 5–30% of cells, as 2 with 31–70% of cells and as 3 with ≥71% of cell. The staining intensity was scored similarly, with 0 used for negative staining, 1 for weakly positive, 2 for moderately positive and 3 for strongly positive. The scores for the percentage of positive tumor cells and for the staining intensity were added to generate an immunoreactive score for each specimen. The product of the quantity and intensity scores were calculated such that a final score of 0–3 indicated weak expression, 4–6 indicated strong expression. Each sample was examined separately and scored by two pathologists.

### Nude mouse tumor xenograft model and CCR10 shRNA treatment

LN229 cells that were cotransducted with CCR10 shRNA, shRNA resistant version of CCR10 and scramble shRNA (from GenePharma, Shanghai, China) and luciferase lentivirus were injected into the intracranial of 5-week-old BALB/c-nu mice[6]. At day 5, 15 30, tumors were measured by fluorescent images of whole mice using an IVIS Lumina Imaging System (Xenogen). After death, the tumor tissues were used for IHC assay for CCR10, p-Akt (S473), Ki-67 and MMP9.

### Statistical analysis

The TCGA mRNA expression microarray data (AgilentG4502A_07_2 array N=483) and metadata including survival information for GBM patients were downloaded from the following portal: http://tcga-data.nci.nih.gov/tcga/homepage.htm. Rembrandt data was downloaded from the following portal: https://caintegrator.nci.nih.gov/rembrandt/home.do. To quantify the visual pattern, Wilcoxon rank-sum test is used to measure the significance of differential gene expression between normal brain tissue and GBM in TCGA and Unpaired t test in Rembrandt. Overall survival curves were plotted according to the Kaplan–Meier method, with the log-rank test applied for comparison. All differences were considered statistically significant at the level of P < 0.05. Statistics were performed using the SPSS Graduate Pack 11.0 statistical software (SPSS, Chicago, IL, USA).

## SUPPLEMENTARY MATERIAL FIGURES AND TABLE


