# Supplementary material for: Upregulation of chemokine receptor CCR10 is essential for glioma proliferation, invasion and patient survival
Source: Oncotarget. 2014 Jun 26;5(16):6576–83. doi: 10.18632/oncotarget.2134 (PMC4196146; doi:10.18632/oncotarget.2134)
Supplement: Supplementary file 1 [file oncotarget-05-6576-s001.pdf]

Upregulation of chemokine receptor CCR10 is essential for glioma proliferation, invasion and patient survival

Supplementary Material

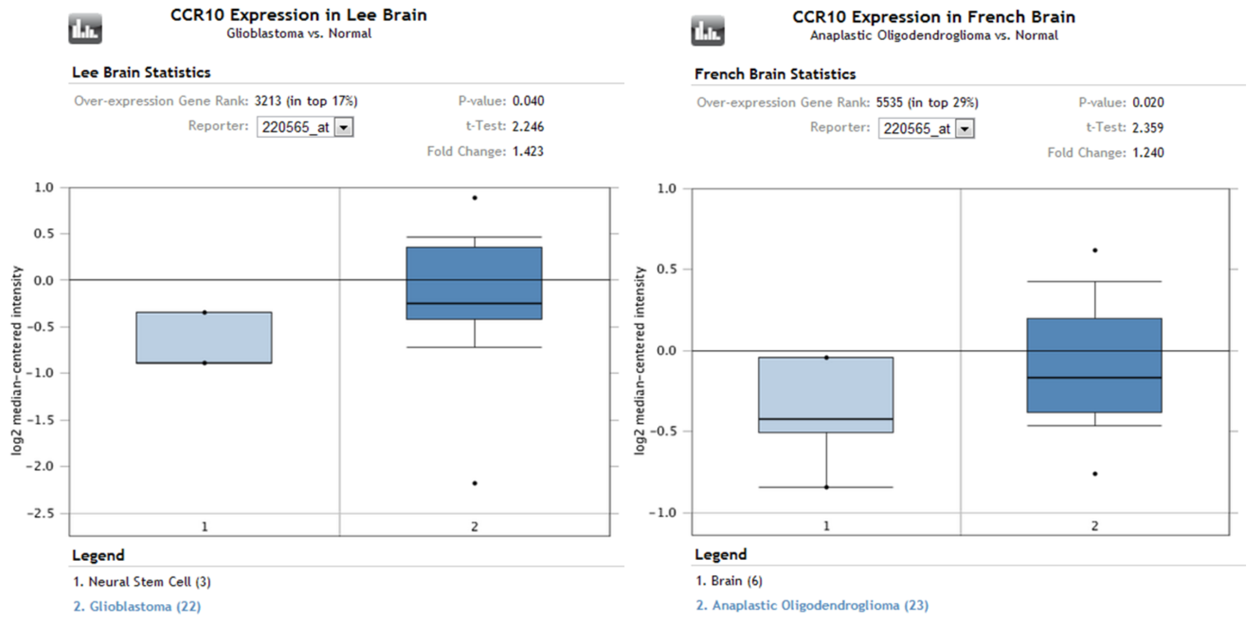

Supplementary Figure 1: The mRNA expression of CCR10 in control brain and GBM in oncomine.

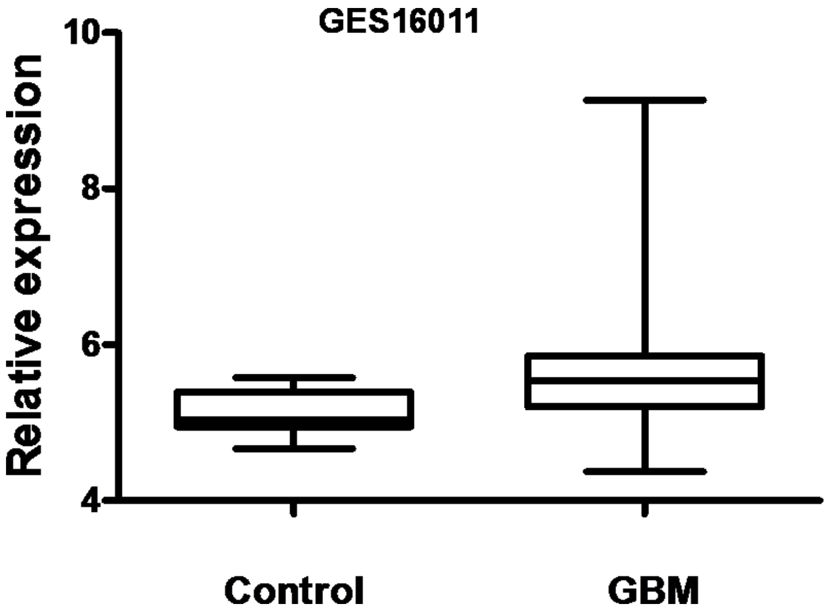

Supplementary Figure 2: The mRNA expression of CCR10 in control brain and glioma in GES16011.

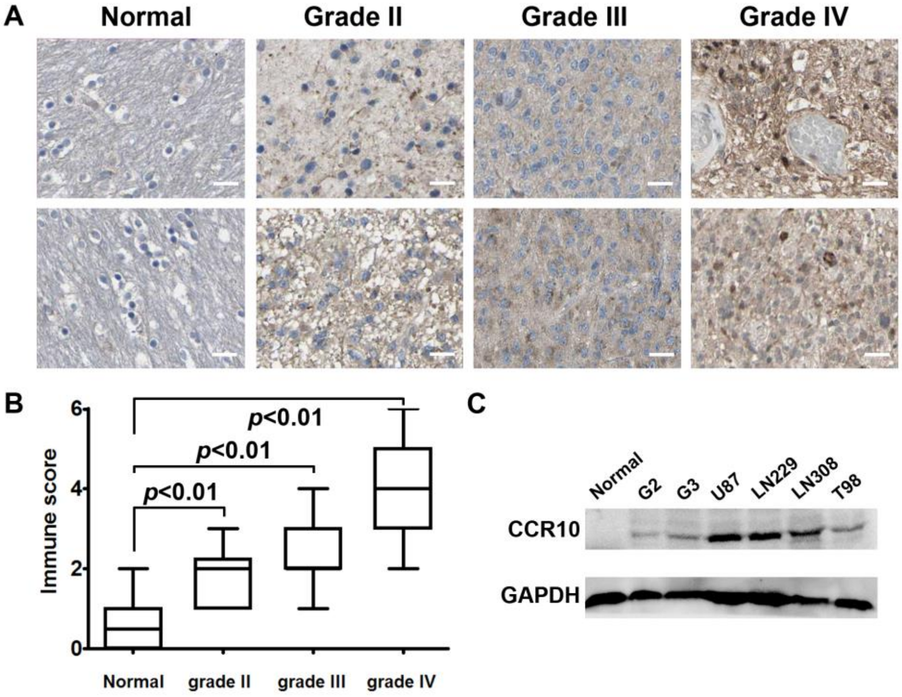

Supplementary figure 3. The expression of CCR10 in different grade glioma (A) Immunohistochemistry with CCR10 antibody on formalin-fixed parafin-embedded brain tissue and different grade glioma samples. (B) Box plot showing significantly higher CCR10 protein expression in high-grade glioma samples than low grade glioma and control brain tissue. (C) Western blot detection of CCR10 in different grade glioma cell lines and control brain tissue. G2: Primitive culture grade II glioma cell. G3: Primitive culture grade III glioma cell.

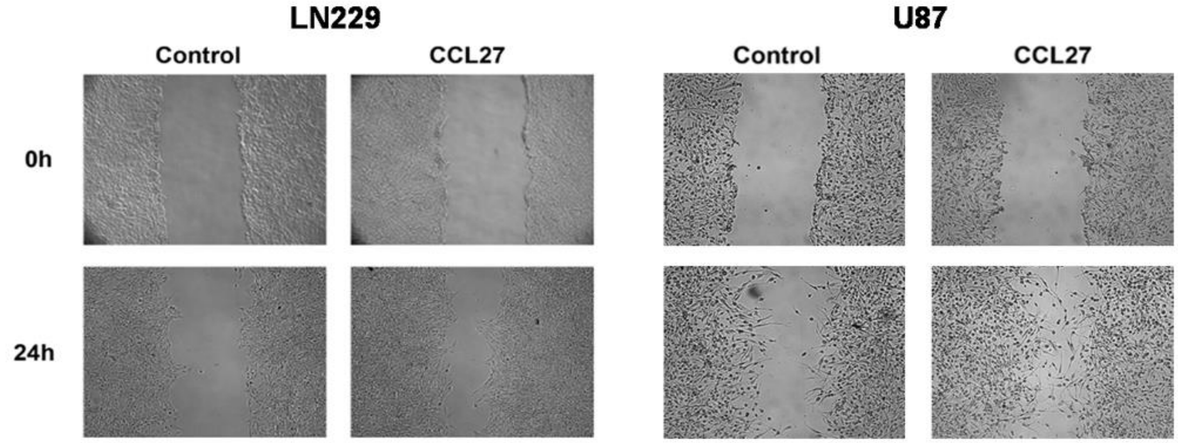

Supplementary figure 4; CCR10 activation promote invasion *in vitro*. Wound healing assays indicate increased invasion in U87 and LN229 cells following CCL27 treatment.

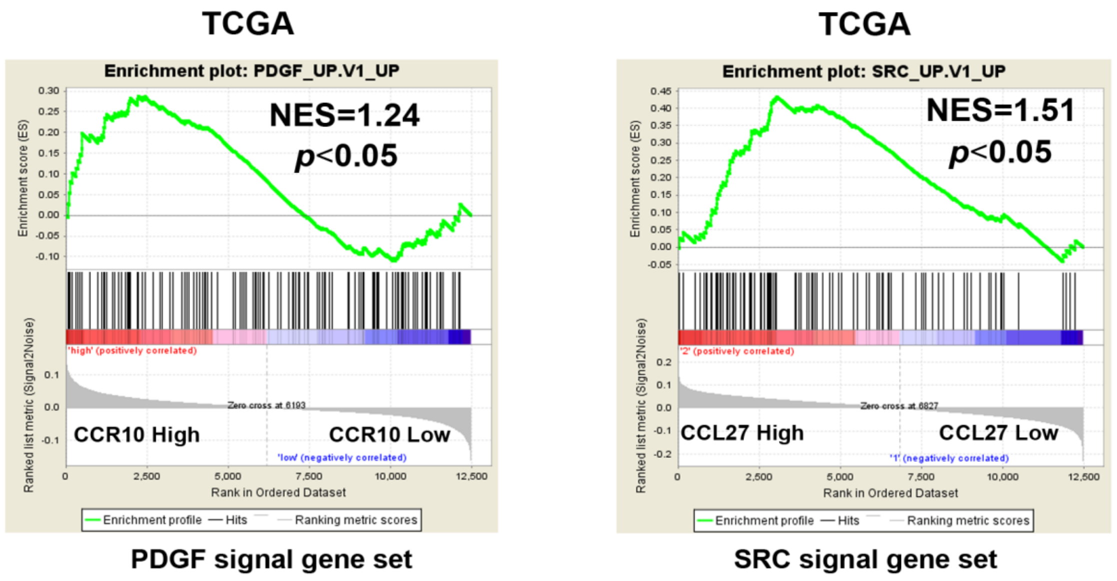

Supplementary figure 5: PDGF and SRC gene set enrichment The enrichment plots of gene expression signatures of PDGF and SRC gene set according to the differences between the samples with high and low CCR10 and CCL27 expression in TCGA data.

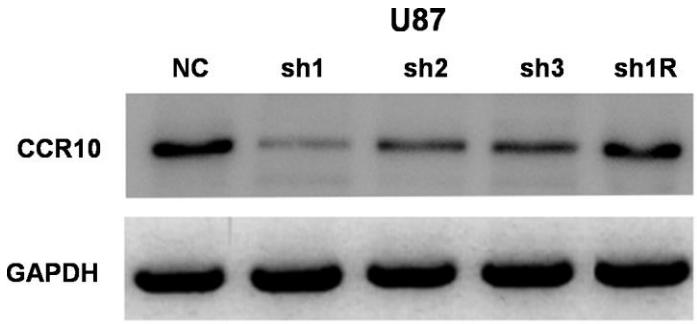

Supplementary figure 6: CCR10 expression was quantified by western blot assay. CCR10 expression significantly decreased by CCR10 siRNA (si-1, si-2, si-3) transfection, relative to the scramble and RNAi resistant version of CCR10.

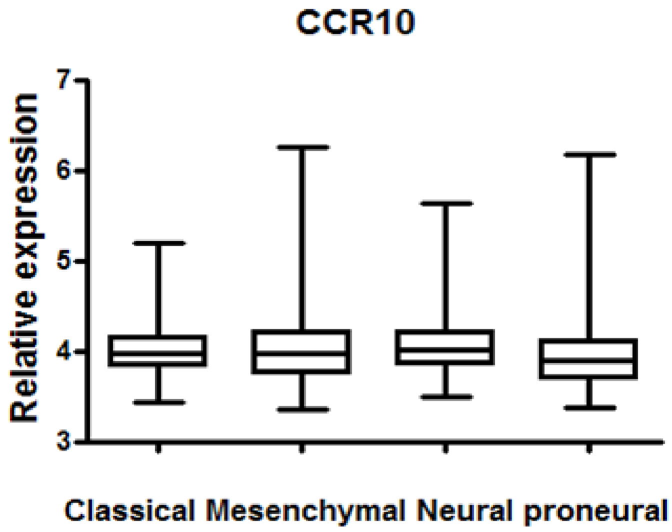

Supplementary figure 7: The expression of CCR10 in different gene subtype GBM. Box plot showing significantly lower CCR10 protein expression in proneural GBM than other subtypes GBM.

Supplementary Table1: Associations of CCR10 with clinicopathologic variables

		CCR10		
		High	Low	
variables	N	N(%)	N(%)	*P*
Sex				
Male	42	18(42.9)	24(57.1)	
Femal	18	13(72.2)	5(27.8)	0.05
Age				
<55	28	18(64.3)	10(35.7)	
≥55	32	13(40.6)	19(59.4)	0.077
P53				
High	27	17(63)	10(37)	
Low	33	14(42.4)	19(57.6)	0.129
Ki-67				
High	23	11(47.8)	12(52.2)	
Low	37	20(54.1)	17(58.6)	0.791
